# Palm Oil Derived Tocotrienol-Rich Fraction Attenuates Vascular Dementia in Type 2 Diabetic Rats

**DOI:** 10.3390/ijms232113531

**Published:** 2022-11-04

**Authors:** Sohrab A. Shaikh, Rajavel Varatharajan, Arunachalam Muthuraman

**Affiliations:** Pharmacology Unit, Faculty of Pharmacy, AIMST University, Semeling, Bedong 08100, Kedah, Malaysia

**Keywords:** tocotrienol-rich fraction, acetylcholinesterase, cerebrovascular disease, homocysteine, morris water maze, platelet-derived growth factor-c, superoxide dismutase

## Abstract

Vascular dementia (VaD) is a serious global health issue and type 2 diabetes mellitus (T2DM) patients are at higher risk. Palm oil tocotrienol-rich fraction (TRF) exhibits neuroprotective properties; however, its effect on VaD is not reported. Hence, we evaluated TRF effectiveness in T2DM-induced VaD rats. Rats were given a single dose of streptozotocin (STZ) and nicotinamide (NA) to develop T2DM. Seven days later, diabetic rats were given TRF doses of 30, 60, and 120 mg/kg orally for 21 days. The Morris water maze (MWM) test was performed for memory assessment. Biochemical parameters such as blood glucose, plasma homocysteine (HCY) level, acetylcholinesterase (AChE) activity, reduced glutathione (GSH), superoxide dismutase (SOD) level, and histopathological changes in brain hippocampus and immunohistochemistry for platelet-derived growth factor-C (PDGF-C) expression were evaluated. VaD rats had significantly reduced memory, higher plasma HCY, increased AChE activity, and decreased GSH and SOD levels. However, treatment with TRF significantly attenuated the biochemical parameters and prevented memory loss. Moreover, histopathological changes were attenuated and there was increased PDGF-C expression in the hippocampus of VaD rats treated with TRF, indicating neuroprotective action. In conclusion, this research paves the way for future studies and benefits in understanding the potential effects of TRF in VaD rats.

## 1. Introduction

Dementia is recognized by the gradual fall in cognition leading to a reduced ability to act independently [1]. The rate of dementia is rapidly rising, and it is one of the main causes of disability [2]. By 2050, there could be 152 million more people with dementia worldwide [3]. According to most studies, vascular dementia (VaD) is the second most common type of dementia [4,5]. There is growing evidence demonstrating a causal relationship between diabetic mellitus (DM) and cognitive impairment [6]. Studies carried out over time revealed that compared to people without DM, those with DM have a higher risk of developing dementia, particularly vascular dementia [7]. Oxygen and glucose must be continuously supplied to the brain, and metabolic processes must be tightly controlled. It has been suggested that the loss of this metabolic control contributes to the memory impairment brought on by neurodegeneration [8]. Type 2 diabetes mellitus (T2DM) makes the neurovascular unit of the brain more vulnerable. Ageing, metabolic interactions and hyperinsulinemia–insulin resistance, oxidative stress, inflammation, and vascular damage are five primary overlapping linkages affecting the neurovascular system [9]. According to clinical and experimental research, long-term hyperglycemia, especially in type 2 diabetes, results in a steady decline in brain neuronal function. Because diabetes affects cerebral vascular supply, common central nervous system (CNS) problems associated with the disease include stroke and cerebral ischemia [10]. There are very few pharmacological treatments for VaD, such as memantine and acetylcholinesterase inhibitors, which may have a moderate benefit in VaD. According to studies, oxidative stress is positively linked to cognitive impairment, and antioxidative substances (beta-carotene, lycopene, vitamin A, vitamin c, alpha-naphtho flavone, and vitamin E) are reported to have a good impact on both cognition and dementia [11,12,13,14].

Antioxidants are considered as a parameter of the protection mechanisms against dementia [15], and natural antioxidants have the role in preventing dementia [16]. Preclinical studies using natural compounds such as resveratrol [17], lycopene [18], and curcumin [19] are reported to have a neuroprotective effect in VaD; moreover, the herbal drug *Ginkgo Biloba* extract is used in the treatment of VaD patients [20], thus indicating the possible role of natural antioxidant in VaD treatment. Furthermore, the synthetic drugs commonly used in the treatment of VaD are cholinesterase inhibitors, which offer minimal benefits at the cost of adverse effects [20]. Hence, in this context, there is an urgent need to identify a suitable drug for the treatment of VaD, perhaps a natural antioxidant.

Palm oil, one of the most produced and consumed edible oils in the world is a good source of beta carotene and vitamin E [13,21], and is extracted from the fruit of the palm tree (*Elaeis guineensis*) [22]. Tocotrienol-rich fraction (TRF) is an isolated fraction of palm oil that consists mainly of a mixture of α, γ, δ-tocotrienols, and some α-tocopherols [23,24]. Moreover, 30% tocopherols and 70% tocotrienols make up palm oil’s vitamin E content while other commonly used dietary vegetable oils contain mainly tocopherols [25,26]. Tocotrienol’s therapeutic effects are extensively reviewed and are reported to have antioxidant, anti-diabetic, nephroprotective, neuroprotective, anti-cancer, anti-osteoporosis, gastroprotective, hepatoprotective, cardioprotective, immunoregulatory, lipid-lowering and anti-inflammatory effects [27,28,29,30]. Moreover, recent studies showed TRF’s potential in managing diabetes and assisting in wound healing by promoting the platelet-derived growth factor-BB (PDGF-BB) in a timely manner, which is important for clean wound closure [31,32]. However, in our literature search, we found that the role of TRF in T2DM-induced VaD remains to be explored. Hence, we hypothesized that TRF treatment in T2DM-induced VaD in rats may act as a neuroprotective agent by attenuating diabetes-induced oxidative stress, cerebrovascular and cholinergic dysfunction, and memory loss. Additionally, we hypothesized that TRF may stimulate platelet-derived growth factor-C (PDGF-C), a potent neuroprotective factor that can rescue neurons from T2DM-induced apoptosis. Therefore, the objective of this study was to evaluate the effect of palm-oil-derived TRF in T2DM-induced VaD in rats by performing behavioral, biochemical, histopathology, and immunohistochemistry studies.

## 2. Results

### 2.1. Effect of TRF on Body Weight

The CON group rats showed an increase in final body weight, while the T2DM-induced group rats showed a decrease in final body weight. There was a highly significant decrease in the final body weights of rats in the T2DM (*p* < 0.001), TRF 30 (*p* < 0.001), TRF 60 (*p* < 0.01), TRF 120 (*p* < 0.01) and DON (*p* < 0.001) groups compared to the CON group. The weight reduction in T2DM-induced rats indicated the development of diabetes. However, T2DM rats treated with TRF 60 and TRF 120 showed a significant (*p* < 0.05) increase in final body weight compared to T2DM group rats, indicating attenuation of T2DM-induced weight loss. Moreover, T2DM rats treated with TRF 30 and DON group showed an increase in final body weight compared to T2DM rats; however, statistically, it was not significant (Table 1).

### 2.2. Effect of TRF on Fasting Blood Glucose

**Figure 1:**
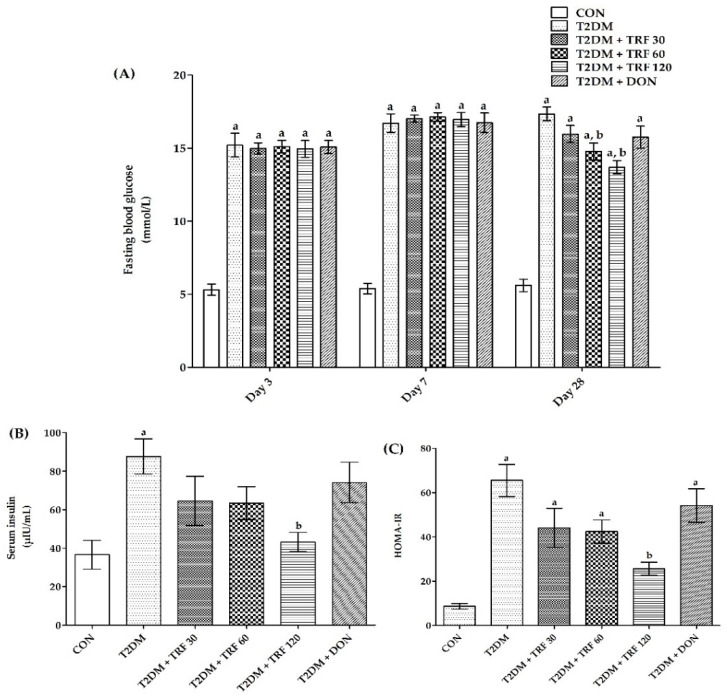
Effect of TRF on FBG (**A**), serum insulin (**B**), and HOMA-IR index (**C**) in T2DM-induced VaD rats. Each group (*n* = 6) represents mean ± SEM. a = *p* < 0.05 vs. CON, b = *p* < 0.05 vs. T2DM. Abbreviations: CON, control; T2DM, type 2 diabetes mellitus; TRF 30, tocotrienol-rich fraction 30 mg/kg; TRF 60, tocotrienol-rich fraction 60 mg/kg; TRF 120, tocotrienol-rich fraction 120 mg/kg; and DON, donepezil.

There was an increase in fasting blood glucose (FBG) after the administration of a single dose of STZ and NA. Rats in T2DM, TRF 30, TRF 60, TRF 120, and DON groups showed a highly significant (*p* < 0.001*)* increase in FBG on days 3, 7, and 28 compared to the CON group, indicating the diabetic condition throughout the study period. However, on day 28, rats treated with TRF 60 showed a significant decrease (*p* < 0.05), and the TRF 120 group showed a highly significant (*p* < 0.001) decrease in FBG compared to the T2DM group, indicating attenuation of FBG in type 2 diabetic rats. Conversely, the TRF 60 and 120 treatments were not effective enough to bring down the raised FBG to normal levels. Moreover, TRF 30 and DON group rats showed a non-significant decrease in FBG compared to the T2DM group (Figure 1A).

### 2.3. Effect of TRF on Serum Insulin

The serum insulin was found to be increased in the type 2 diabetic rats. The rats in the T2DM group showed a highly significant (*p* < 0.01) increase in serum insulin compared to the CON group, indicating the development of hyperinsulinemia. However, rats treated with TRF showed a dose-dependent decrease in serum insulin but only TRF 120 showed a significant (*p* < 0.05) decrease in serum insulin compared to T2DM group rats. This effect of TRF indicates attenuation of hyperinsulinemia in type 2 diabetic rats. Moreover, DON group rats showed a non-significant decrease in FBG compared to the T2DM group (Figure 1B).

### 2.4. Effect of TRF on Homeostatic Model Assessment for Insulin Resistance (HOMA-IR)

The HOMA-IR was found to be increased in the type 2 diabetic rats. The T2DM rats showed a highly significant (*p* < 0.001) increase in the HOMA-IR index compared to the CON group, indicating the development of insulin resistance in type 2 diabetic rats. However, rats treated with TRF showed a dose-dependent decrease in HOMA-IR but only TRF 120 showed a highly significant (*p* < 0.001) decrease in HOMA-IR index compared to the T2DM group rats, indicating attenuation of insulin resistance in type 2 diabetic rats. Moreover, DON group rats showed a non-significant decrease in the HOMA-IR index compared to the T2DM group (Figure 1C).

### 2.5. Effect of TRF on T2DM Induced Changes on Escape Latency Time (ELT) and Time Spent in Target Quadrant (TSTQ), Using Morris Water Maze (MWM)

**Figure 2:**
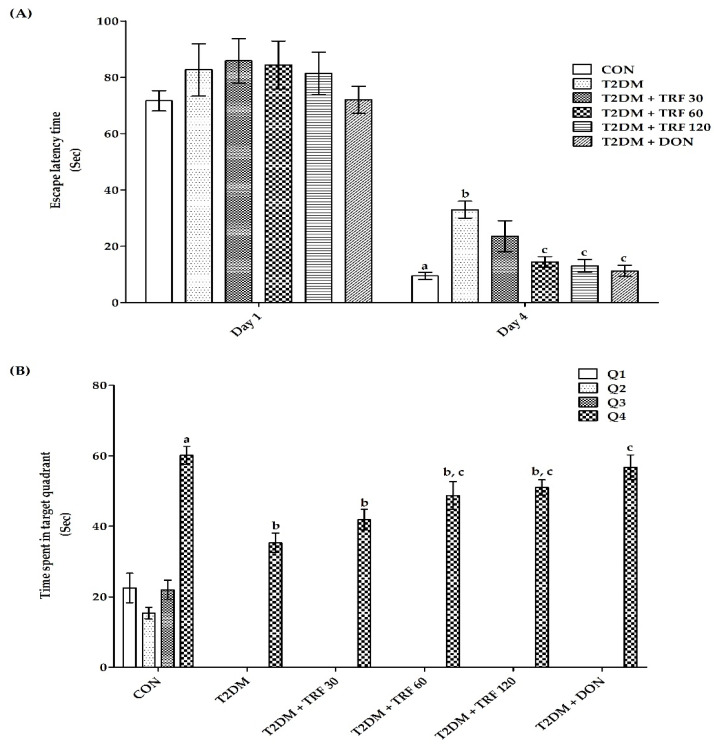
Effect of TRF in T2DM-induced VaD rat on day 4 escape latency time (**A**) and day 5 time spent in the target quadrant (**B**) using Morris Water Maze test. Each group (*n* = 8) represents mean ± SEM. a = *p* < 0.05 vs. day 1 ELT of CON, b = *p* < 0.05 vs. day 4 ELT of CON, c = *p* < 0.05 vs. day 4 ELT of T2DM (Figure 2A). a = *p* < 0.05 time spent in Q1, Q2, Q3 vs. Q4 quadrant in CON. b = *p* < 0.05 vs. time spent in target quadrant Q4 of CON, c = *p* < 0.05 vs. time spent in target quadrant Q4 of T2DM (Figure 2B). Abbreviations*:* CON, control; T2DM, type 2 diabetes mellitus; TRF 30, tocotrienol-rich fraction 30 mg/kg; TRF 60, tocotrienol-rich fraction 60 mg/kg; TRF 120, tocotrienol-rich fraction 120 mg/kg; and DON, donepezil.

**ELT**: The CON group rats showed a highly significant (*p* < 0.001) decrease in day 4 ELT compared to day 1 ELT, indicating normal learning ability. Further, there was a highly significant (*p* < 0.01) increase in day 4 ELT in T2DM group rats compared to day 4 ELT in the CON group, indicating impairment of acquisition. However, rats treated with TRF 60, TRF 120, and DON showed a significant (*p* < 0.05) decrease in day 4 ELT compared to T2DM group rats, indicating attenuation of T2DM-induced impairment of acquisition (Figure 2A).

**TSTQ:** The CON group rats showed a highly significant (*p* < 0.001) increase in TSTQ (Q4) compared to time spent in other quadrants, reflecting normal memory retrieval behavior. Further rats in T2DM, TRF 30, and TRF 60 showed a highly significant (*p* < 0.001 and *p* < 0.01) decrease in TSTQ compared to TSTQ of the CON group; similarly, TRF 120 group rats showed a significant (*p* < 0.05) decrease in TSTQ, indicating T2DM induced impairment of memory. However, rats treated with TRF 60, TRF 120, and DON showed a highly significant (*p* < 0.001) increase in TSTQ compared to T2DM group rats TSTQ, indicating attenuation of T2DM-induced impairment of memory (Figure 2B).

### 2.6. Effect of TRF on Plasma Homocysteine Levels

The rats in T2DM and TRF 30 groups showed a highly significant (*p* < 0.001) increase in the plasma homocysteine levels compared to the CON group, indicating T2DM-induced hyperhomocysteinemia. However, rats treated with TRF 60, TRF 120, and DON showed a highly significant (*p* < 0.001) decrease in the plasma homocysteine levels, indicating attenuation of T2DM-induced hyperhomocysteinemia (Table 2).

### 2.7. Effect of TRF on Brain Acetylcholinesterase (AChE) Activity

The rats in T2DM and TRF 30 groups showed a highly significant (*p* < 0.001) and significant (*p* < 0.05) increase in the brain AChE activity, respectively, compared to the CON group, indicating T2DM-induced cholinergic dysfunctioning. However, rats treated with TRF 60, TRF 120, and DON showed a highly significant (*p* < 0.001) decrease in the brain AChE activity, indicating attenuation of T2DM-induced cholinergic dysfunctioning (Table 2).

### 2.8. Effect of TRF on Reduced Glutathione (GSH) Levels

The rats in the T2DM group showed a highly significant (*p* < 0.001) decrease in the brain GSH levels compared to the CON group, indicating T2DM-induced elevation of brain oxidative stress. However, rats treated with TRF 60, TRF 120, and DON showed a highly significant (*p* < 0.01 and *p* < 0.001) increase in the brain GSH levels compared to the control group, indicating attenuation of T2DM-induced brain oxidative stress (Table 2).

### 2.9. Effect of TRF on Total Superoxide Dismutase (T-SOD) Activity

The rats in the T2DM group showed a highly significant (*p* < 0.01) decrease in the brain T-SOD levels compared to the CON group, indicating T2DM-induced elevation of brain oxidative stress. However, rats treated with TRF 60 showed a significant (*p* < 0.05) increase, while TRF 120 and DON showed a highly significant (*p* < 0.01) increase in the brain T-SOD levels compared to the control group, indicating attenuation of T2DM-induced brain oxidative stress (Table 2).

### 2.10. Effect of TRF on Histopathological Changes in the Hippocampus

**Figure 3:**
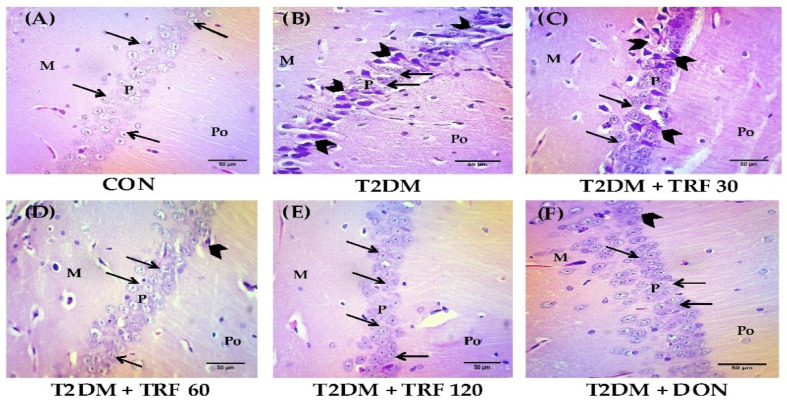
Photomicrograph representing H&E stained CA1 area of the hippocampus. (400× magnification) scale bar 50 µm. CON group rat shows normal molecular (M), pyramidal (P), and polymorphic (Po) layers with neurons intact and properly arranged having a circular shape and lightly stained as shown by arrows in the P layer. Neurodegeneration was not evident and normal histology was observed (**A**). The T2DM-induced VaD rat group shows abnormal M, P, and Po layers with presences of vacuolations, pyknotic pyramidal cells (as shown by arrowhead), and disorganized P layer (**B**). T2DM-induced VaD rat treated with TRF shows dose-dependent improvement in M, P, and Po layers with a smaller number of pyknotic cells and a properly arranged P layer (**C**–**E**). T2DM-induced VaD rat treated with DON shows a normal structure in M and Po layers with no pyknotic cells, however, the P layer appears to be disorganized (**F**). Abbreviations: CON, control; T2DM, type 2 diabetes mellitus; TRF 30, tocotrienol-rich fraction 30 mg/kg; TRF 60, tocotrienol-rich fraction 60 mg/kg; TRF 120, tocotrienol-rich fraction 120 mg/kg; and DON, donepezil.

The brain coronal section stained with H&E stain was observed under a light microscope for studying the histopathological changes in the hippocampus. Neurons in the hippocampal cornu ammonis 1 (CA1) region are susceptible to oxidative stress and vulnerable to injury induced by oxidative stress [33]. Hence, histopathological studies were conducted in the CA1 region of the hippocampus. The CON group showed normal structure with no damaged neurons in CA1 of the hippocampus. There was a compact cellular structure of stratum pyramidale, clear cytoplasm, and distinct nucleus; nuclei were large, round, and lightly stained. In contrast, the T2DM VaD rat’s neurons were irregular and loosely arranged and showed a condensed nucleus. Neurodegeneration was observed by intense purple staining in the elongated irregular nucleus and shrunken cytoplasm, i.e., karyopyknotic neurons were observed; moreover, only a few cells had a viable nucleus, and the cell density had drastically reduced with increased intercellular space, which indicates massive neuronal damage. However, TRF dose-dependent treatment alleviated the structural changes in T2DM-induced VaD rats, TRF preserved the compact structure of stratum pyramidale and prevented loss of neuronal cells with fewer pyknotic nuclei, which showed less neuronal cell damage. Similar changes were also observed in DON-treated rats; however, neurons were slightly irregular and loosely arranged (Figure 3).

### 2.11. Effect of TRF on PDGF-C Expression in the Hippocampus

**Figure 4:**
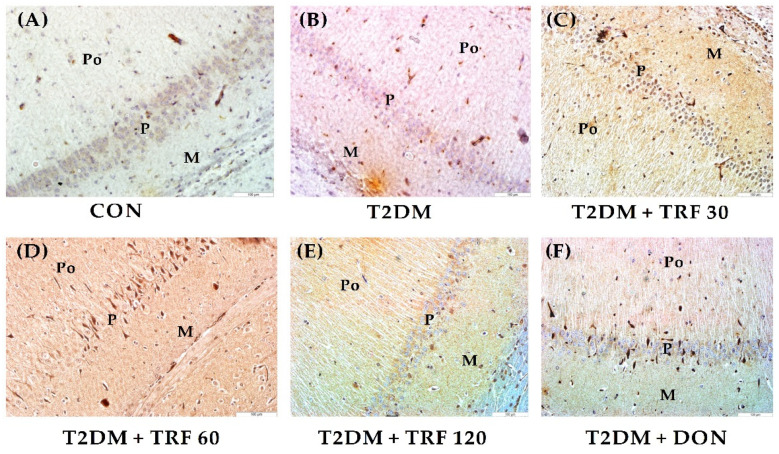
Photomicrograph representing immunohistochemical staining of PDGF-C expression in the CA1 area of the hippocampus. (200× magnification) scale bar 100 µm. CON group rat stained faintly positive for PDGF-C expression (**A**). T2DM-induced VaD group rat stained negative for PDGF-C expression (**B**). TRF 30 group rat stained mid-positive, indicating PDGF-C expression in molecular (M), polymorphic (Po), and pyramidal (P) layers (**C**). TRF 60 group rat stained mid to strong positive, indicating high PDGF-C expression in M, Po, and P layers (**D**)**.** TRF 120 group rat stained mid-positive indicating PDGF-C expression in M, Po, and P layers (**E**). DON group rat stained faintly positive for PDGF-C expression (**F**). Abbreviations: CON, control; T2DM, type 2 diabetes mellitus; TRF 30, tocotrienol-rich fraction 30 mg/kg; TRF 60, tocotrienol-rich fraction 60 mg/kg; TRF 120, tocotrienol-rich fraction 120 mg/kg; and DON, donepezil.

The CON group rats showed positive immunostaining in polymorphic, molecular, and pyramidal layers, indicating the normal conserved PDGF-C expression in the CA1 area of the hippocampus. In contrast, T2DM-induced VaD rats showed negative staining, indicating the lack of PDGF-C expression, perhaps due to T2DM-induced neuronal apoptosis. However, TRF 30, 60, and 120 treatments in T2DM rats showed positive immunostaining in the CA1 area. Notable high immunostaining in polymorphic, molecular, and pyramidal layers was observed with TRF 60 treatment. This effect of TRF indicates its possible role in stimulating PDGF-C to attenuate T2DM-induced neurovascular changes in VaD rats. Moreover, the DON group showed low immunostaining compared to the TRF group (Figure 4).

## 3. Discussion

The most prevalent kind of diabetes, T2DM, is characterized by hyperinsulinemia and insulin resistance and is known to increase the chance of dementia [34,35]. Insulin resistance impairs neural plasticity, metabolism, and cell survival while promoting apoptosis, oxidative stress, and cytokine activation [36,37]. We studied the neuroprotective effects of TRF in T2DM-induced vascular dementia in rats. T2DM was induced by administering a single dose of STZ and NA. Numerous studies have shown that the STZ and NA diabetes model is valuable in investigations of various aspects of diabetes since STZ is known to damage pancreatic B-cells, while NA is given to rats to partially protect insulin-secreting cells against STZ [38,39]. In the present study, T2DM was confirmed in rats by the increased levels of fasting blood glucose, the condition called hyperglycemia [40], and it was constant in T2DM rats throughout the study. Additionally, we found a significant body weight reduction in T2DM rats, which is one of the signs of diabetes induction; similar findings were also reported earlier with STZ and NA models [41,42]. Moreover, there was an increased insulin level observed in the T2DM rats that signified the development of insulin resistance. However, we confirmed the development of insulin resistance in T2DM by HOMA-IR values, which is a convenient and beneficial method for evaluating insulin resistance [43]. In this work, T2DM rats displayed hyperglycemia, hyperinsulinemia, and severe insulin resistance that likely hampered cell survival, metabolism, and neural plasticity while boosting oxidative stress, cytokine activation, and apoptosis and causing rats to develop VaD [36]. TRF 60 and 120 treatment was able to attenuate the hyperglycemia, hyperinsulinemia, and high insulin resistance in T2DM rats, indicating its potential to manage T2DM; however, the attenuation was not effective enough to bring the glucose levels back to normal. Our findings are in agreement with previous studies indicating TRF effects in controlling T2DM conditions [44,45,46,47,48]. Importantly, we observed a reduction in insulin resistance, demonstrating the role of TRF in enhancing the effects of insulin. Because insulin is thought to have neuroprotective properties and exert neurotrophic effects on CNS neurons [49], this action of TRF is advantageous in reducing the risk of developing VaD. Moreover, our findings are in agreement with previous studies indicating TRF’s role in increasing insulin sensitivity by modulating peroxisome proliferator-activated receptors (PPAR) [50,51] and regulating the insulin signaling pathway in hyperglycemia [52,53]. Conversely, DON has not shown a significant effect in controlling FBG and insulin resistance.

Further, the Morris water maze test was conducted to assess memory functioning in T2DM-induced VaD rats. The Morris water maze (MWM) is widely used to evaluate rodent spatial learning and memory [54] that relies on the time taken by animals to find the platform, i.e., escape latency as a parameter to quantify spatial memory and learning [55,56]. Further, animals are subjected to a probe trial, which is usually conducted 24 h after the final acquisition trial to assess memory retrieval. For the probe trial, the most frequent parameter used is time spent in the target quadrant compared to the other quadrants [57]. A longer time spent in the target quadrant reflects a superior retrieval memory [58]. In the present study, we found that T2DM VaD rats displayed an increased escape latency time compared to normal rats, indicating reduced spatial learning and memory throughout the task. Moreover, T2DM VaD rats also spent less time in the target quadrant compared to the other quadrants and normal rats. However, TRF (60 and 120 mg/kg) treatment in T2DM VaD rats was able to significantly attenuate the loss of spatial acquisition and memory retrieval, perhaps by protecting the neurons of the hippocampus and preventing memory loss. Further, our findings are also in agreement with previously published studies that reported administration with TRF enhances spatial learning as measured by the Morris water maze task [25,59,60,61]. Moreover, TRF (60 and 120 mg/kg) effects were found to be as effective as DON, perhaps by decreasing the AChE activity.

It is widely known that the pathophysiology of memory loss during T2DM-induced encephalopathy involves degraded cholinergic function in multiple brain areas [62]. Acetylcholine (ACh) plays a significant role in cholinergic neuron functioning in the hippocampus and controls the learning and memory process [63]. In the cholinergic system, choline acetyltransferase (ChAT) and AChE are involved in the synthesis and metabolism of ACh, respectively; however, STZ administration increases AChE activity [64]. The increased AChE activity leads to decreased ACh concentration that affects cognitive functioning [65]. In our study, we found that T2DM rats showed a significant rise in AChE activity, indicating cholinergic dysfunctioning that would have caused cognitive impairment in diabetic rats. However, TRF (60 and 120 mg/kg) treatments in T2DM rats attenuated the increased AChE activity and improved the cholinergic functioning, and protected the cognitive impairment, perhaps by inhibiting AChE activity and increasing ACh concentration. Our findings about AChE are following the previous studies that have reported tocotrienol showing the inhibition of acetylcholinesterase activity [66,67,68,69]. Therefore, we suggest that TRF exhibits anticholinesterase activity that can be essential for improving cholinergic function in VaD rats. Moreover, TRF (60 and 120 mg/kg) effects were found to be comparable with the standard treatment drug DON. Therefore, we suggest that TRF may have exhibited anticholinesterase activity that is essential for improving cholinergic function in VaD rats.

Further, one of the main goals of preventing dementia in T2DM is preventing vascular damage [70]. T2DM is an independent risk factor for macrovascular and microvascular complications [71]. One known condition to affect cerebrovascular homeostasis is hyperhomocysteinemia (HHcy) and evidence suggests that HHcy may promote dementia by more than one mechanism, including cerebral microangiopathy, endothelial dysfunction, oxidative stress, neuronal damage, enhancement of beta-amyloid peptide-mediated vascular toxicity, neurotoxicity, and apoptosis [72,73,74,75,76]. In patients with diabetes, elevated homocysteine (HCY) levels were associated with insulin resistance [77] and poor endothelial function [78]. Moreover, cognitive status in type 2 diabetes is correlated with homocysteine levels [79]. It is also reported that an elevated level of plasma total homocysteine is an important risk factor for VaD [80], and is an important blood biomarker in VaD diagnosis [72]. In the present study, we found that T2DM rats showed HHcy, which may have accounted for cerebrovascular dysfunction. However, the HHcy in T2DM rats was attenuated by TRF 60 and 120 treatments. Our findings are in agreement with previous studies that have reported palm TRF in reducing high-methionine diet-induced hyperhomocysteinemia [81,82] possibly by reducing hepatic oxidative stress and possibly by exerting a direct inhibitory effect on hepatic cystathionine β-synthase [83]. Hence, TRF’s significant effect in reducing the increased levels of plasma homocysteine would have improved cerebrovascular functioning to overcome endothelial dysfunction and cerebral angiopathy in T2DM-induced VaD rats. Moreover, DON treatment also attenuated HHcy in T2DM rats, and the findings are following previous studies with DON [84]

Further, when the antioxidant defense is compromised due to oxidative stress, functions are disrupted and cells are damaged, which results in the loss of synapses and cell death. Free radical production and lipid peroxidation are promoted by STZ in the brain [85]. Multiple signaling pathways are activated by hyperglycemia increasing the generation of ROS [86]. In the current work, the antioxidant capacity parameters SOD and GSH were investigated in T2DM-induced VaD rats. We found that T2DM rats displayed decreased levels of brain GSH and SOD, indicating hyperglycemia-induced oxidative stress, which is responsible for the activation of different pathways responsible for inducing potential vascular damage [10]. In our study, TRF (60 and 120 mg/kg) treatments in T2DM VaD rats were able to ameliorate the oxidative stress by increasing the GSH and SOD levels; this effect further shows its potential as an antioxidant in the brain to act as neuroprotective. Our findings are also in agreement with a previous study that reported TRF antioxidant activity [44,45,46,50,87,88]. TRF perhaps by interrupting free radical chain reactions and by capturing the free radical would have imparted antioxidant activity [50] in the brain to attenuate the T2DM-induced oxidative stress. Therefore, the TRF antioxidant activity detected in our study would have provided the maximum protection of neurons in T2DM-induced VaD rats from the deleterious effects of oxidative stress. Moreover, DON treatment also displayed significant antioxidant activity by increasing GSH and SOD levels; this finding of DON antioxidant activity is following previously reported studies [84,89,90,91]. According to earlier studies, donepezil appears to have beneficial effects on redox homeostasis. AChE inhibition also appears to have a significant antioxidant role and may prevent protein oxidation by reducing AChE activity [89].

Furthermore, in the present study, we observed significant histopathological changes in T2DM rats. In both rats and humans, the hippocampus is a crucial structure that is linked to learning and memory. Therefore, the pathological basis of cognitive impairment in diabetic rats may be due to the changes in the hippocampus’s structure caused by hyperglycemia [92,93], and the earlier report states that type 2 diabetes is associated with accelerated cognitive decline and structural brain abnormalities [94]. Thus, we hypothesized that T2DM-induced oxidative stress can cause structural changes in the hippocampus. The brain coronal sections stained with H&E displayed vacuolization around the neuron or perinuclear space, disrupting degenerated neurons, and the cytoplasm of neurons in the T2DM group of rats was shrunken and stained dark in CA1 areas of the hippocampus. However, TRF treatment was able to alleviate the T2DM-induced structural changes of neurons in the hippocampus; perhaps this effect of TRF is due to its antioxidant property that prevented the neuron from being damaged. Our results are consistent with earlier research using TRF, which showed that rats given vitamin E had a larger number of viable pyramidal cells [95]. The hippocampal neuronal structure protection provided by TRF further reinforces its neuroprotective property. Moreover, DON treatment was able to alleviate the T2DM-induced structural changes of neurons; however, there was disorganization of neurons in the pyramidal layer of the hippocampus CA 1 area.

The vascular and nervous systems share many anatomical features, and there are molecular similarities between these two systems. Platelet-derived growth factors (PDGFs) play a crucial role in angiogenesis [96], and their member PDGF-C is expressed in the neurovascular unit in human brains, playing an essential role in the formation of normal cerebral vascularization [97]. According to reports, PDGF plays a role in the development of diabetes-related vascular complications, particularly PDGF-C [96]. The actions of PDGFs vary across a wide range of cell types, and PDGF-C promotes angiogenesis and revascularizes ischemic tissues, after an ischemic insult, PDGF therapy is said to increase blood flow while simultaneously protecting neurons from degeneration [96]. Importantly, studies suggest that the management of ischemic stroke in mice involves the use of PDGF-C and its receptor PDGFR, which are “drugable” targets. This is probably because PDGF-C is necessary for the development of normal cerebral vascularization, and cerebral ventricles, and for maintaining the integrity of the neuro-ependymal tissue [97]. Furthermore, studies have demonstrated that PDGF-CC inhibition reduces pathological angiogenesis by having an impact on multiple cellular and molecular targets [98]; however, PDGF-C expression in pathological conditions is essential for neovascularization. Additionally, PDGF-C-based studies appear to be conducted specifically to promote its angiogenic, proliferative, neuroprotective, and anti-apoptotic properties [99]. Hence, we hypothesized that TRF may stimulate PDGF-C release that can rescue neurons from T2DM-induced apoptosis in VaD rats. To prove this hypothesis, immunohistochemical staining of the hippocampus was performed for analyzing PDGF-C expression. We found that T2DM rats showed decreased PDGF-C expression compared to the control group. Our findings are in agreement with earlier reports indicating that PDGF-C expression is down-regulated in ischemic tissues in a mouse model of diabetes, resulting in angiogenic impairment [96]. However, T2DM rats treated with TRF showed increased PDGFC expression, indicating that TRF may have stimulated the endogenous PDGF-C a neuroprotective factor to rescue neurons from T2DM-induced apoptosis [99]. This IHC study finding can be correlated with recent studies indicating the role of TRF in promoting PDGF-BB in the blood during the initial wound healing stage and producing clean wound closure [31,32], indicating that TRF has a role in stimulating endogenous PDGF in pathological conditions or injuries. Hence, we suggest that TRF has a potential role in enhancing the PDGF-C and protecting the neuron from neurodegeneration. Moreover, this action of TRF to stimulate endogenous PDGFC could induce angiogenesis and revascularization of ischemic tissues and be involved in the formation of normal cerebral vascularization to augment the blood flow; however, further study with TRF is warranted in this regard. Moreover, our findings with DON treatment in T2DM rats showed that PDGF-C expression was less expressed compared to TRF treatment.

## 4. Materials and Methods

### 4.1. Animals

Sprague-Dawley male rats weighing around 160–210 gms were obtained from AIMST University central animal house. The animal study protocol was approved by the AIMST University Animal Ethics Committee (AUAEC/FOP/2020/12). Rats were housed in AIMST University central animal house under standard laboratory conditions. Rats were fed with a normal pellet diet, with free access to fresh water. Rats were maintained at 22 ± 1 °C and 12 h light:dark cycle was followed. All the experiments were performed during the light period. Before experimentation, animals were allowed to become accustomed to the surrounding conditions.

### 4.2. TRF Drug Preparation

TRF used in this research work was Gold Tri.E™ 70 (Liquid) received as a gift sample from Sime Darby Oils Nutrition Sdn Bhd. Malaysia. Gold Tri.E 70 is a liquid mixture of tocotrienols, and tocopherols extracted from palm fruit (*Elaeis Guineensis*). TRF was dissolved in olive oil (Basso, Italy) to prepare three different doses (30, 60, and 120 mg/kg/bw) [53,81] before oral administration in rats.

### 4.3. Induction of T2DM-Induced Vascular Dementia

Type 2 Diabetes mellitus (T2DM) was induced in overnight fasted rats by injecting a single dose of STZ (50 mg/kg; *i.p*) 15 min after NA (120 mg/kg; *i.p*. mg/kg, *i.p.*) administration [100,101]. Before administration of STZ, STZ was dissolved in 0.1 M citrate buffer and NA was dissolved in saline. Streptozotocin (STZ) was obtained from Merck KGaA, Darmstadt, Germany and nicotinamide (NA) from Sigma-Aldrich, St. Louis, MO, USA. Inclusion and exclusion criteria: rats with fasting glucose levels ≥ 13 mmol/L [62] on the third and seventh day after streptozotocin injection were considered T2DM animals and were included in the experimental protocol; however, non-diabetic rats were excluded.

### 4.4. Animal Grouping and Experimental Design

A total of six groups were made for the animal study consisting of 9 rats in every single group. All the animals were kept on a normal pellet diet and received the treatment as per the respective group dosing schedules.

Group I: Control (CON): Rats in this group served as normal control rats without any treatment.

Group II: Type 2 diabetes mellitus (T2DM): Rats in this group served as type 2 diabetes mellitus rats without any treatment.

Group III: Type 2 diabetes mellitus + Tocotrienol-rich fraction 30 (T2DM + TRF 30): T2DM rats in this group were treated with tocotrienol-rich fraction (30 mg/kg; *p.o.*) from day 8 to day 28.

Group IV: Type 2 diabetes mellitus + Tocotrienol-rich fraction 60 (T2DM+ TRF 60): T2DM rats in this group were treated with tocotrienol-rich fraction (60 mg/kg; *p.o.*) from day 8 to day 28.

Group V: Type 2 diabetes mellitus + Tocotrienol-rich fraction 120 (T2DM+ TRF 120): T2DM rats in this group were treated with tocotrienol-rich fraction (120 mg/kg; *p.o.*) from day 8 to day 28.

Group VI: Type 2 diabetes mellitus + Donepezil (T2DM+ DON): T2DM rats in this group were treated with donepezil (1 mg/kg; *p.o.*) [102] from day 8 to day 28.

### 4.5. Body Weight

Individual animal’s initial body weight on day 3 of the study i.e., after T2DM induction and final body weight on day 28 of the study were measured. Rats were placed individually on the digital weighing machine for measuring body weight. The mean change in body weight was calculated.

### 4.6. Morris Water Maze Test

Rat’s learning and memory were evaluated using the Morris water maze (MWM) test [103]. The MWM test is based on the concept that since animals do not like to swim, they would try to get out of the water by locating a platform [57]. MWM test was performed by using a circular water pool (150 cm in diameter; 45 cm in height). Further, the water pool was divided into 4 quadrants, quadrant 1 (Q1), quadrant 2 (Q2), quadrant 3 (Q3), and quadrant 4 (Q4); moreover, the different visual cues on the inner wall of each quadrant were pasted. A submerged platform (10 × 10 cm and 28 cm in height) was placed in the middle of the target quadrant (Q4) of this pool; later, the water was filled up to the level of 2 cm above the platform. The position of the platform was kept unchanged throughout the training session. The water maze task was carried out for four consecutive days from days 24–27. The rats received four consecutive daily training sessions in the following 4 days (day 24 of the study was considered as day 1 of training while day 27 of the study was considered as day 4 of training), with each trial having a cut-off time of 120 s. Escape latency time (ELT) to locate the hidden platform in MWM was noted as an index of acquisition or learning. Once the animals found the platform, they were allowed to stay on the platform for 15 s. If animals failed to find the platform, they were guided to the platform after 120 s. The probe test was carried out on the fifth day (28th day of study), the platform was removed, water was made opaque by adding milk powder and each animal individually was placed in the middle of the pool and allowed to swim for 120 s. The time spent in the target quadrant (TSTQ), i.e., Q4, by each animal was recorded. TSTQ was considered as a parameter to analyze an animal’s ability to retain memory after the removal of the platform [57,104].

### 4.7. Blood Sample Collection and Brain Homogenate Preparation

On the last day of the study, rats were individually anesthetized using ether by inhalation method. Blood was withdrawn by the retroorbital puncture method. The plasma and serum were separated and stored at −80 °C until the time of biochemical estimations. Immediately after the blood withdrawal, the rats were sacrificed individually by cervical dislocation. The rat brains were isolated, and the brain weight was recorded. The whole brain of a rat was further homogenized in phosphate buffer (pH 7.4). Further, the homogenate was centrifuged at 3000 rpm for 15 min to obtain clear supernatant. The supernatant was separated and stored at −80 °C until the time of biochemical estimations. Additionally, for histopathology and immunohistochemistry studies, two brain samples from each group were collected and immersed in 10% neutral buffered formalin.

### 4.8. Blood Glucose Measurement

The fasting blood glucose (FBG) levels in rats were measured on days 3, 7, and 28 of the study periods. Blood was withdrawn from the rat tail vein, and blood glucose was measured using a glucometer (Accu-chek Active, Roche) according to the manufacturer’s instructions. The FBG results are expressed as mmol/L.

### 4.9. Serum insulin and Homeostatic Model Assessment for Insulin Resistance (HOMA-IR)

Serum insulin was measured using a rat insulin ELISA Kit (Wuhan Fine Biotech Co., Ltd. Wuhan, China) according to the manufacturer’s instructions. The results obtained are expressed as µIU/mL. Further, HOMA-IR was used to identify insulin resistance. HOMA-IR was calculated using the following formula [105,106].

HOMA-IR = [fasting glucose (mmol/L) × fasting insulin (µIU/mL)]/22.50

### 4.10. Plasma Homocysteine

Plasma homocysteine levels were determined by using rat homocysteine (HCY) ELISA Kit (SunLong Biotech Co., LTD, Zhejiang, China). The analysis was conducted according to the manufacturer’s instructions. The results are expressed as µmol/L.

### 4.11. Acetylcholinesterase Activity

For the estimation of acetylcholinesterase activity, Ellman’s method was used [107]. The acetylcholinesterase activity was measured by using acetylthiocholine (ATC) as an artificial substrate. Thiocholine released because of the cleavage of ATC by AChE was allowed to react with the Ellman’s reagent 5,5′- dithiobis-(2-nitrobenzoic acid) (DTNB), which was reduced to thionitrobenzoic acid, a yellow-colored anion with absorption maxima at 412 nm. The concentration of thionitrobenzoic acid was detected using a UV spectrophotometer (Shimadzu UV 1800) and taken as a direct estimate of the AChE activity. Briefly, 0.4 mL of brain homogenate was added to a cuvette containing 2.6 mL of phosphate buffer (0.1 M, pH 7.4) and 100 μL of DTNB. The contents of the cuvette were mixed thoroughly and the changes in absorbance were measured using a UV visible spectrophotometer (UV-1800 Shimadzu Spectrophotometer, Shimadzu Corporation, Kyoto, Japan) at 412 nm wavelength. When absorbance reached a stable value, it was recorded as the basal reading. Further, 20 μL of ATC was added and a change in absorbance was recorded at intervals of 2 min. Changes in absorbance per min were calculated by using the following formula [108,109,110] and the results are expressed as µmol/min/gm of tissue.

R = 5.74 × 10^−4^ × ∆*A/CO*

R = Rate in moles of substrate hydrolyzed/min/g tissue; Δ*A* = Change in absorbance/min; *CO* = Original concentration of the tissue (mg/mL).

### 4.12. Reduced Glutathione

The reduced glutathione (GSH) content in tissue was estimated using the method of Beutler et al. [111]. Briefly, 0.5 mL of brain homogenate was mixed with 2 mL of 0.3 M disodium hydrogen phosphate. Later, 0.25 mL of 0.001 M freshly prepared DTNB (dissolved in 1% *w*/*v* sodium citrate) was added. The changes in absorbance were recorded using a UV visible spectrophotometer (UV-1800 Shimadzu Spectrophotometer, Shimadzu Corporation, Kyoto, Japan) at 412 nm wavelength. To estimate the sample GSH concentration, a standard curve was plotted using 10–100 µM of the reduced form of glutathione. The results of GSH are expressed as µmol/mg of protein. The brain’s total protein was determined by Lowry’s method [112].

### 4.13. Superoxide Dismutase Activity

T-SOD levels were determined by using a superoxide dismutase activity assay kit (Elabscience Biotechnology Inc, Houston, Texas, USA). The analysis was conducted on rat whole brain homogenate according to the manufacturer’s instructions. The results are expressed as units/mg of protein.

### 4.14. Histopathological Study

The coronal sections of the rat brain samples were stained with hematoxylin and eosin (H&E) stain for histopathological study. The H&E-stained slides were observed under the Digital Binocular Microscope at 400× magnification for analyzing the structural changes in the hippocampus. The rat brain coronal section and staining procedure were performed by Gribbles Pathology Lab, Sungai Petani, Kedah, Malaysia.

### 4.15. Immunohistochemistry (IHC) for Identification of Platelet-Derived Growth Factor-C (PDGF-C) Expression in Rat Brain Hippocampus

The IHC study was performed by Eman Biodiscoveries Sdn. Bhd., Sungai Petani, Kedah, Malaysia. IHC optimized protocol (Abcam, USA) was followed. The primary antibody (Rabbit Polyclonal against Platelet-Derived Growth Factor C (PDGFC), Abbexa-UK) and secondary antibody (Rabbit specific HRP_DAB IHC Detection Kit—Micro-polymer v2d, ab236469, Abcam, USA) was used. The method was performed according to manufacturer (Abcam) instructions. The slides were observed under the microscope for identifying the intensity of immunostaining in the hippocampus for PDGF-C expression.

### 4.16. Statistical Analysis

Data obtained from behavioral tests were statistically analyzed by applying a two-way analysis of variance (ANOVA) followed by a Bonferroni post-test. The data obtained from the biochemical evaluation were statistically analyzed using one-way ANOVA followed by Tukey’s multiple comparison test. All data were analyzed using GraphPad Prism version 5.03 software. The results were expressed as mean ± standard error of means (SEM) and the probability value of *p* < 0.05 was considered to be statistically significant.

## 5. Conclusions

In the present study, we found that palm oil derived TRF exhibits a significant effect in reducing insulin resistance, protecting memory loss, improving cerebrovascular functioning, enhancing cholinergic activity, reducing neuronal damage by exerting antioxidant activity, protecting the hippocampal structure, and notably enhancing the PDGF- C expression for neovascularization in the diabetic brain. Additionally, the TRF 60 and 120 effects were found to be comparable with standard drug DON; thus, it would be interesting to study the combined effects of TRF and DON in VaD. Moreover, based on our study findings, there exists future scope for evaluating TRF effects in different risk factors involved in VaD development. Hence, we conclude that palm oil derived TRF possesses neuroprotective effects in T2DM-induced VaD rats. Moreover, this study finding puts forward new knowledge about palm oil derived TRF’s role in the management of vascular dementia.

## Figures and Tables

**Table 1:** Effect of TRF on T2DM-induced changes in VaD rat’s body weight.

Treatment	Initial Body Weight (gms)	Final Body Weight (gms)
CON	202.50 ± 3.62	214.30 ± 3.33
T2DM	203.30 ± 3.42	186.30 ± 2.46 ^a^
T2DM + TRF 30	203.80 ± 4.10	190.00 ± 3.63 ^a^
T2DM + TRF 60	205.90 ± 2.52	198.10 ± 1.58 ^a,b^
T2DM + TRF 120	204.60 ± 2.91	199.00 ± 2.38 ^a,b^
T2DM + DON	205.00 ± 3.38	191.60 ± 2.47 ^a^

Each group (*n* = 8) represents mean ± SEM. ^a^ = *p* < 0.05 vs. control, ^b^ = *p* < 0.05 vs. T2DM. Abbreviations: CON, control; T2DM, type 2 diabetes mellitus; TRF 30, tocotrienol-rich fraction 30 mg/kg; TRF 60, tocotrienol-rich fraction 60 mg/kg; TRF 120, tocotrienol-rich fraction 120 mg/kg; and DON, donepezil.

**Table 2:** Effect of TRF on plasma homocysteine, brain acetylcholinesterase activity, brain reduced-glutathione, and total superoxide dismutase levels in T2DM induced VaD rats.

Treatment	Plasma Homocysteine (µmol/L)	Brain AChE Activity (µmol /min/gm of Tissue)	Brain GSH (µmol/mg of Protein)	Brain T-SOD (Units/mg of Protein)
CON	4.72 ± 0.66	2.17 ± 0.17	8.49 ± 0.33	24.48 ± 1.09
T2DM	12.92 ± 0.68 ^a^	4.54 ± 0.39 ^a^	5.72 ± 0.34 ^a^	14.60 ± 1.08 ^a^
T2DM + TRF 30	11.34 ± 0.53 ^a^	3.69 ± 0.38 ^a^	6.65 ± 0.21	18.05 ± 1.11
T2DM + TRF 60	7.24 ± 0.68 ^b^	2.42 ± 0.34 ^b^	8.55 ± 0.43 ^b^	22.88 ± 2.63 ^b^
T2DM + TRF 120	6.56 ± 0.51 ^b^	2.15 ± 0.19 ^b^	9.71 ± 0.60 ^b^	24.75 ± 2.05 ^b^
T2DM + DON	7.14 ± 0.57 ^b^	2.12 ± 0.27 ^b^	9.95 ± 0.59 ^b^	23.83 ± 1.24 ^b^

Each group (*n* = 6) represents mean ± SEM. ^a^ = *p* < 0.05 vs. CON; ^b^ = *p* < 0.05 vs. T2DM. Abbreviations: CON, control; T2DM, type 2 diabetes mellitus; TRF 30, tocotrienol-rich fraction 30 mg/kg; TRF 60, tocotrienol-rich fraction 60 mg/kg; TRF 120, tocotrienol-rich fraction 120 mg/kg; and DON, donepezil.

## Data Availability

The data presented in this study are available on request from the corresponding author.
